# Expression of the innate immune receptor LILRB5 on monocytes is associated with mycobacteria exposure

**DOI:** 10.1038/srep21780

**Published:** 2016-02-24

**Authors:** Louise E. Hogan, Des C. Jones, Rachel L. Allen

**Affiliations:** 1 Institute for Infection and Immunity, St George’s, University of London, Cranmer Terrace, London, SW17 0RE; 2 TB Research Group, Animal and Plant Health Agency, Weybridge, New Haw, KT15 3NB, UK; 3 Immunology Division, Department of Pathology, University of Cambridge, Tennis Court Road, Cambridge, CB2 1QP.

## Abstract

Antigen presenting cells (APC) are critical components of innate immunity and consequently shape the adaptive response. Leukocyte Ig Like Receptors (LILR) are innate immune receptors predominantly expressed on myeloid cells. LILR can influence the antigen presenting phenotype of monocytic cells to determine the nature of T cell responses in infections including *Mycobaterium leprae*. We therefore investigated the relevance of LILR in the context of *Mycobacterium tuberculosis*. Real-time PCR studies indicated that the transcriptional profile of the orphan receptor LILRB5 was significantly up-regulated following exposure to mycobacteria. Furthermore, LILRA1 and LILRB5 were able to trigger signalling through direct engagement of mycobacteria using tranfectant cells incorporating a reporter system. We describe for the first time the expression of this receptor on T cells, and highlight the potential relevance to mycobacterial recognition. Furthermore, we demonstrate that crosslinking of this receptor on T cells increases proliferation of cytotoxic, but not helper, T cells.

Leukocyte immunoglobulin-like receptors (LILR) are a group of receptors with activating or inhibitory functions, that are expressed on the surface of myelomonocytic cells, including antigen presenting cell subsets. In addition to their classification as activatory (LILRA1-2, 4–6) or inhibitory (LILRB1-5) on the basis of signalling motifs, LILR are designated as group I or group II on the basis on their predicted ability to bind MHC class I (MHC-I)^1^,^2^. Through their expression on monocytic cells LILR have been shown to regulate TLR activity^3^,^4^,^5^,^6^,^7^,^8^,^9^, antigen presenting phenotype^10^,^11^,^12^,^13^ and cytokine secretion profile^7^,^14^,^15^, thus demonstrating the potential to influence subsequent immune responses^4^,^16^,^17^,^18^. Variations in the strength of binding of LILRB2 to different HLA alleles expressed by HIV infected patients was recently shown to correlate with control of infection^19^.

In *Mycobacterium leprae (M. Leprae)* infection, LILR are thought to be responsible for influencing the immune response and their expression profile has been shown to correlate with disease phenotype. Comparison of lepromatous and tuberculoid leprosy samples showed increased expression of LILRA2 in the skin lesions of patients with lepromatous leprosy, a disease profile associated with disseminated disease and ineffective bacilli clearance resulting from a Th2 biased response. This is in contrast to tuberculoid leprosy, in which a strong Th1 biased response enables the effective clearance of bacilli from localised infection. Crosslinking of LILRA2 on macrophages resulted in a 40% reduction in intracellular killing of mycobacteria, indicating that LILRA2 may inhibit anti-microbial functions in macrophages. The ligation of LILRA2 also considerably reduced the induction of IL-12 production by TLRs, skewing cytokine activity towards a Th2 biased response^4^,^20^.

As there is evidence for LILR-mediated modulation of immune responses during *M. leprae* infection, it is possible that the expression and functions of these receptors are relevant during infection with other mycobacteria such as *Mycobacterium tuberculosis (M. tb)*. In support of this, the up-regulation of HLA-G, the highest affinity self-ligand for LILRB1 and LILRB2, has been documented by both gene expression assays^21^ and surface expression analysis of macrophages from patients with *M. tb*^22^. LILRB1 expression on NK cells correlates with disease severity in pulmonary tuberculosis^23^, while expression on CD4 T cells, together with CD152 has been proposed to regulate cytolytic activity^24^. Furthermore, TLR2/1/6 heterodimers, TLR4, TLR8, and TLR9 and are implicated in the initial recognition of *M.tb*, and the regulation of these receptors may contribute to down-stream responses^25^,^26^,^27^,^28^,^29^. We therefore decided to investigate the potential role of LILR in the context of *M. tb* infection.

Tuberculosis *per se*, primarily results from infection with the organism *M. tb*, but there have been documented cases indicating *Mycobacterium bovis (M. bovis)* as a causative agent^30^,^31^. Furthermore, the attenuated strain of *M. bovis* used in vaccinations, Bacillus Calmette-Guérin (BCG), causes disease in immunocompromised individuals^32^. We therefore hypothesized that LILR responses may vary between mycobacterial species. Furthermore, non-specific protection arising from various live vaccinations is thought to develop through epigenetic changes in the innate immune environment^33^. This led us to consider that prior BCG vaccination may exert a relevant effect on monocytic cell phenotype. To elucidate the mechanisms involved, we sought to compare LILR expression profiles for antigen presenting cells (APC) exposed to *M. tb*, *M. bovis*, and BCG comparing cells from BCG vaccinated and unvaccinated donors.

LILR can interact directly with bacteria, specifically, LILRB1 and LILRB3 have been shown to bind bacteria including *E*. *coli, H. pylori* and *S. aureus*^34^ with individual receptors varying in their bacterial specificity. The bacterial ligand(s) involved in this interaction are yet to be determined in full, although lipoteichoic acid on *S*. *aureus* has been suggested as one potential ligand^35^. Therefore, it is also possible that LILR may directly interact with one or more mycobacterial ligands. As LILR regulate TLR activity, any potential interaction with mycobacterial antigens could have a distinct impact on the resulting immune response.

Here, we describe variations in LILR profile between APC derived from BCG vaccinated and unvaccinated individuals, which indicate that BCG vaccination decreases the expression of LILRA3 and LILRB2 on monocyte-derived macrophages. Moreover, LILRB5 is up-regulated in monocyte-derived DC in response to mycobacterial species, significantly so after BCG exposure. Furthermore, BCG elicits LILRA1 and LILRB5 signalling in a reporter system. Finally, we describe for the first time LILRB5 expression on T cells, and demonstrate that ligation of LILRB5 on T cells induces CD8, but not CD4, T cell proliferation.

## Results

### Comparison of LILR expression profiles for BCG vaccinated and unvaccinated donors

**Figure 1:**
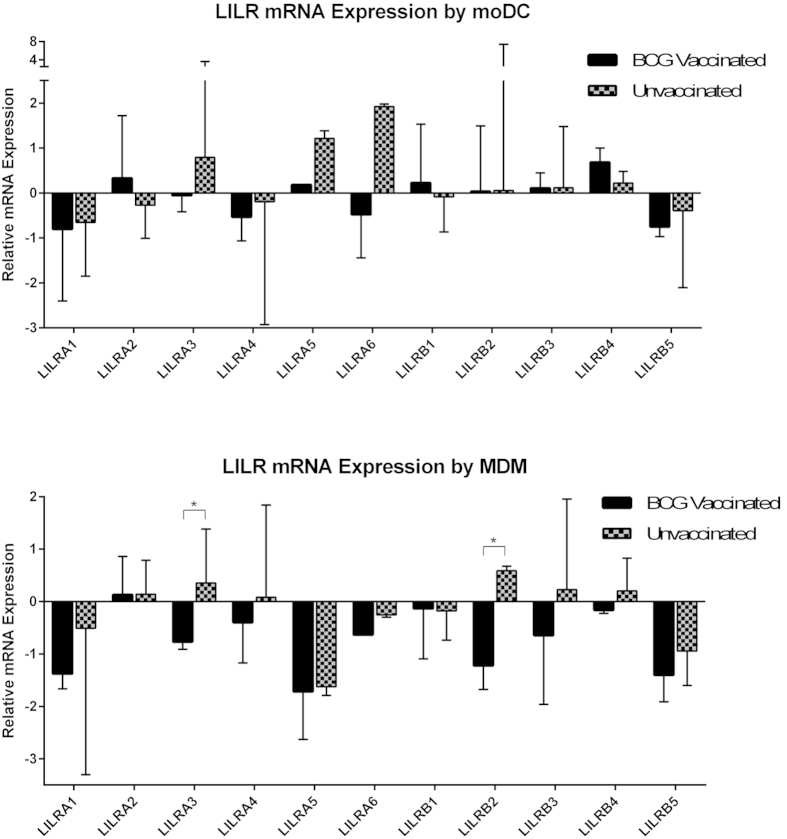
Expression of LILR encoding transcripts in moDC and MDM samples derived from BCG vaccinated and unvaccinated donors, as determined using qPCR analysis. The y axis denotes Ct value, which is relative to GAPDH expression. Mann-Whitney analysis demonstrated a significant difference (p < 0.05) in MDM expression of LILRA3 (p = 0.027) and LILRB2 (p = 0.025) between samples derived from BCG vaccinated and unvaccinated donors. The number of paired samples used for each condition is depicted in Table 1.

To date, LILR expression profile studies have been hindered by a lack of specific monoclonal antibodies for each member of the receptor family. As such, we analysed the 11 human LILR using a Real-Time PCR assay. Distinct mRNA profiles of LILR expression were observed for both *in vitro* monocyte-derived macrophages (MDM) and monocyte-derived DC (moDC) from BCG vaccinated vs. unvaccinated donors (Fig. 1). Statistically significant differences were seen for two of the eleven LILR in MDM. Most strikingly, LILRA3, the only LILR to lack any transmembrane or cytoplasmic domain, had significantly (p = 0.027) lower expression on MDM samples from BCG vaccinated donors compared with unvaccinated donors. LILRB2 was also expressed at lower levels by MDM from vaccinated donors (p = 0.025). As both of these receptors are known to have inhibitory functions, this may be a potentially beneficial finding in the presence of mycobacterial infection.

### BCG vaccination affects LILR expression profiles in the presence of mycobacteria

LILR mRNA expression profiles were then compared for moDC and MDM following *in vitro* culture with BCG, *M. bovis* and *M. tb* (H37Rv). Donors were grouped based on their BCG vaccination status.

**Figure 2:**
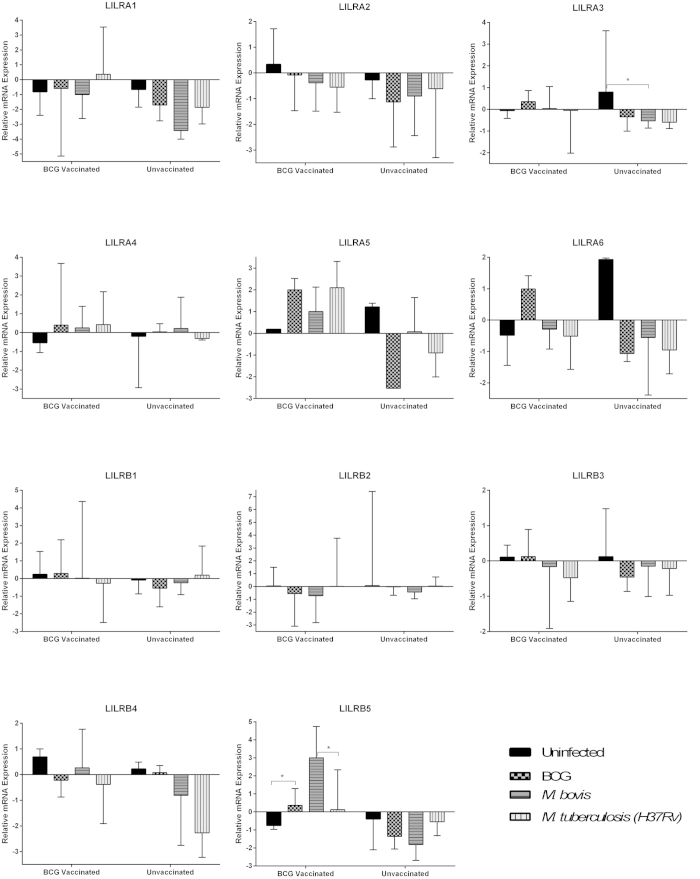
Expression of LILR encoding transcripts in moDC samples derived from BCG vaccinated and unvaccinated donors, cultured with various mycobacterial species, as determined using qPCR analysis. The y axis denotes Ct value, which is relative to GAPDH expression. Mann-Whitney analysis demonstrated a significant difference in moDC expression of LILRA3 between untreated and *M. bovis* treated samples from unvaccinated donors (p = 0.028). Furthermore, LILRB5 demonstrated significant differences between untreated and BCG treated samples from BCG vaccinated donors (p0.034), and *M. bovis* and H37Rv treated samples also from BCG vaccinated donors (p = 0.041). The number of paired samples used for each condition is depicted in Table 1.

In dendritic cell cultures, LILRA3 was down-regulated following culture with all three organisms for moDC derived from unvaccinated donors; however, this down-regulation was only significant following *M. bovis* exposure (p = 0.028) (Fig. 2). These effects were not detected for moDC derived from BCG vaccinated donors. The most striking results were seen for LILRB5, in which moDC derived from BCG vaccinated donors up-regulated LILRB5 following exposure to all three mycobacteria, significantly so following culture with BCG (p = 0.034). These changes in expression were not observed for moDC derived from unvaccinated donors.

**Figure 3:**
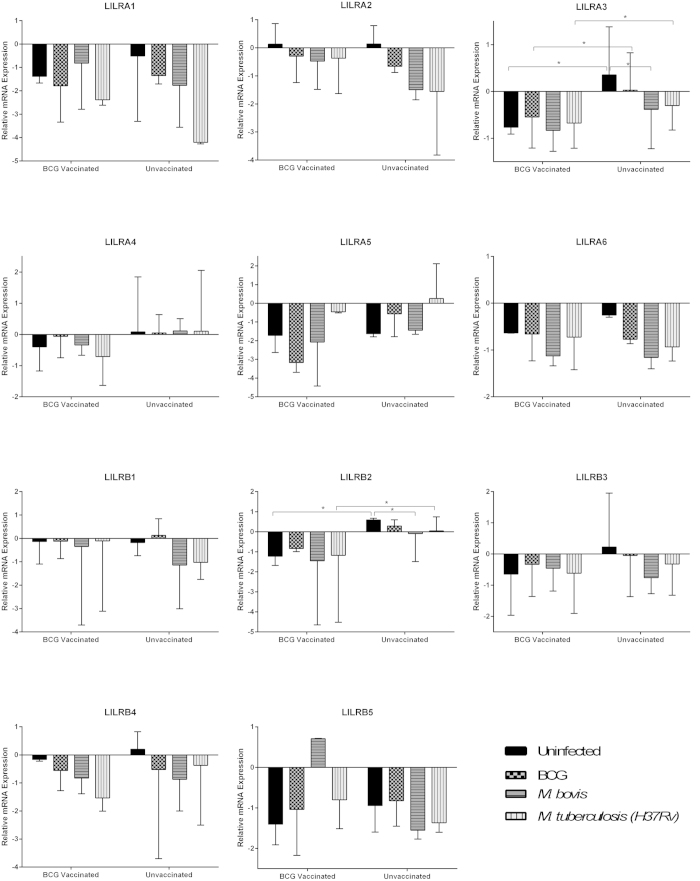
Expression of LILR encoding transcripts in MDM samples derived from BCG vaccinated and unvaccinated donors, cultured with various mycobacterial species, as determined using qPCR analysis. The y axis denotes Ct value, which is relative to GAPDH expression. Mann-Whitney analysis demonstrated a significant difference in MDM expression of LILRA3 between untreated and *M. bovis* treated samples from unvaccinated donors (p = 0.047), and between BCG vaccinated and unvaccinated donors in untreated (p = 0.027), BCG treated (p = 0.047) and H37Rv treated (p = 0.047) samples. Furthermore, LILRB2 demonstrated significant differences between untreated and *M. bovis* treated samples from unvaccinated donors (p0.016), and between BCG vaccinated and unvaccinated donors in untreated (p = 0.025) and H37Rv treated samples (p = 0.028).

For macrophage cultures, LILRA3 was again down-regulated following exposure to *M. bovis* and H37RV, significantly so for *M. bovis* (p = 0.047) in BCG unvaccinated donors (Fig. 3). Furthermore, following culture with BCG and H37Rv, MDM derived from BCG vaccinated donors expressed significantly less LILRA3 compared to their unvaccinated counterparts (BCG: p = 0.047, H37Rv: p = 0.047). LILRB2 was also found to be expressed in significantly higher levels following H37Rv culture on MDM derived from unvaccinated donors, than their BCG vaccinated counterparts (P = 0.028).

It is interesting to note that LILRB5 is the only receptor up-regulated in response to mycobacterial exposure, which may indicate a more distinct role for this receptor in *M. tb* infection. In order to examine the potential relevance of this we proceeded to examine direct binding within a reporter system.

### BCG elicits LILRA1 and LILRB5 signalling in a reporter system

**Figure 4:**
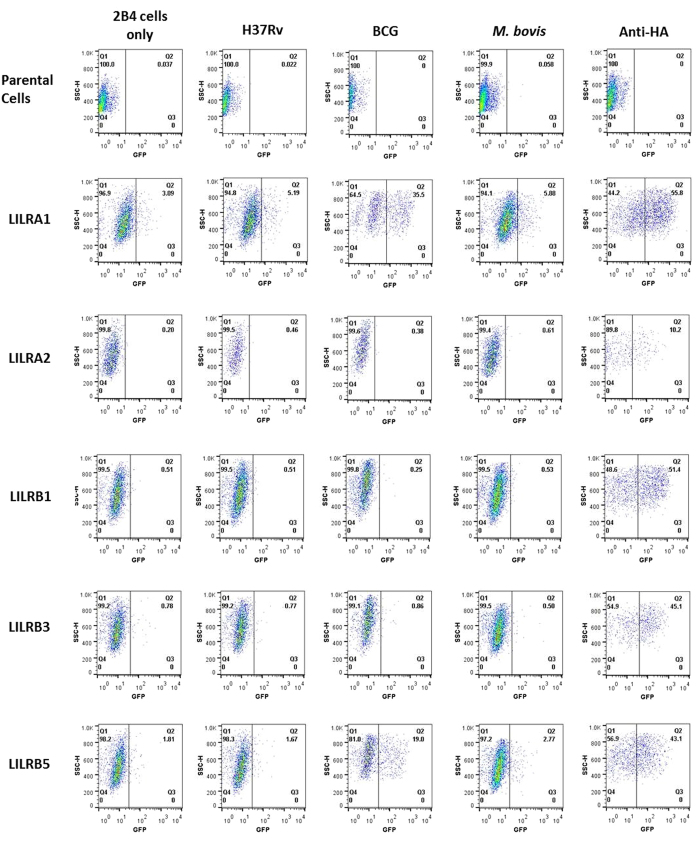
Engagement of mycobacteria by transfected LILR. Detection of GFP production resulting from engagement of LILR with *M. tb*, *M. bovis* or BCG. These are representative results from *n* = 3 experiments, from which all data was consistent.

Binding of whole bacteria by individual LILR and PIR has been demonstrated in previous studies^34^,^35^ with lipoteichoic acid suggested as a potential bacterial ligand for these receptors^35^. It is therefore possible that a subset of LILR could engage components of *M. tb, M. bovis* or BCG through a similar process. To determine whether any members of the LILR family may be capable of direct interaction with mycobacteria, a panel of 2B4 reporter cell lines each expressing an NFAT-GFP (Nuclear Factor of Activated T cell–Green Fluorescent Protein) reporter construct and a LILR-CD3ζ construct were used. As 2B4 is a T cell hybridoma cell line, it would not be expected to internalise mycobacteria. Signalling transfectants of LILRA1, LILRA2, LILRB1, LILRB3, LILRB5 and LILR-negative controls were used to determine whether signalling was induced in the presence of *M. bovis* (heat-killed), *M. tb* (heat-killed) and BCG (live). No equivalent transfectants of LILRA3, LILRA4, LILRA5, LILRA6, LILRB2 or LILRB4 were used (Fig. 4).

The results indicate that BCG can interact with both LILRA1 and LILRB5 to induce signalling through the CD3ζ construct in this system. A slight shift in fluorescence was detectable for LILRA1 transfectants incubated with *M. bovis*, which may indicate a weak interaction. None of the transfected LILR could interact with the H37Rv strain of *M. tb*.

**Figure 5:**
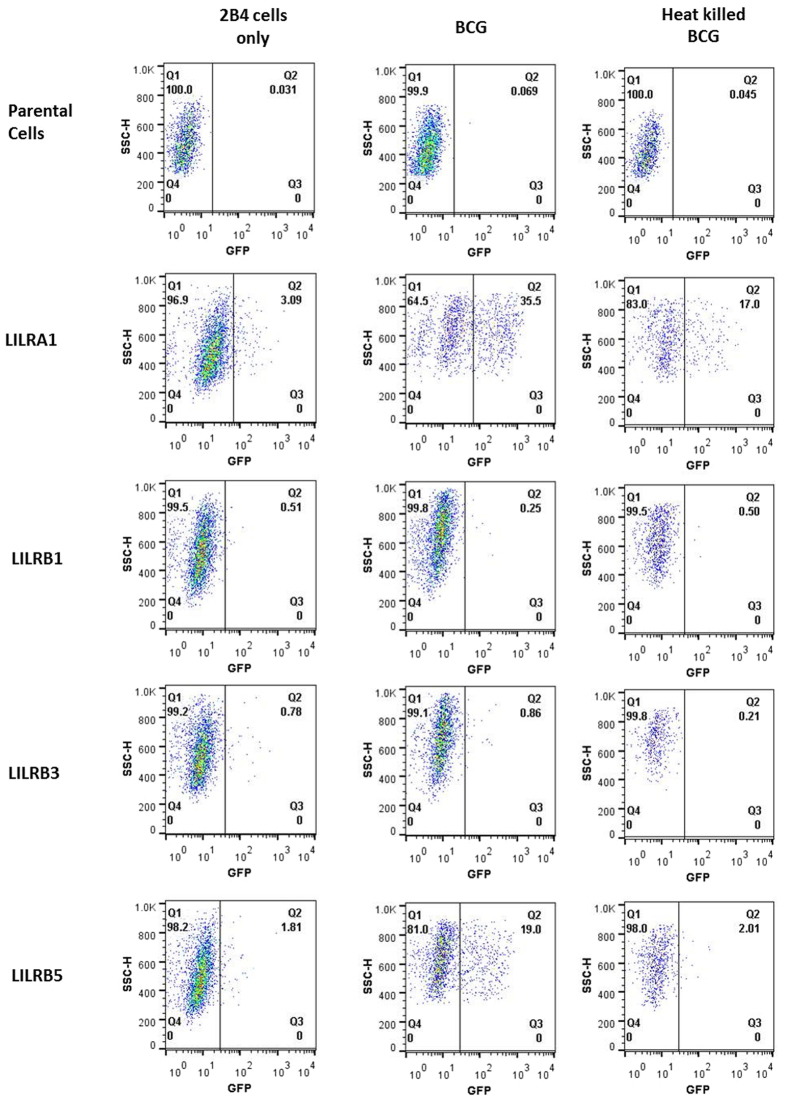
Engagement of BCG and heat killed BCG by transfected LILR. Detection of GFP production resulting from engagement of LILR with heat killed BCG, and compared to live BCG from Fig. 4. These are representative results from *n* = 3 experiments, from which all data was consistent.

Given the difference in results observed between heat-killed *M. bovis* and live BCG, the effect of heat killing on receptor binding was assessed using BCG. To achieve this, BCG was grown to exponential phase and heat killed prior to incubation with the signalling transfectant lines, and the results were compared with live BCG (Fig. 5). LILRB1 and LILRB3 transfectant lines were used as negative controls for this experiment due to their lack of interaction with live BCG. The results demonstrate that heat killing of mycobacteria may reduce receptor binding and consequent signalling, as evidenced by the reduced signalling observed for LILRB5 incubated with heat killed mycobacteria. Thus, the reduction in binding seen after heat killing may indicate that the ligand(s) is not heat stable.

### LILRB5 is expressed on T cells

**Figure 6:**
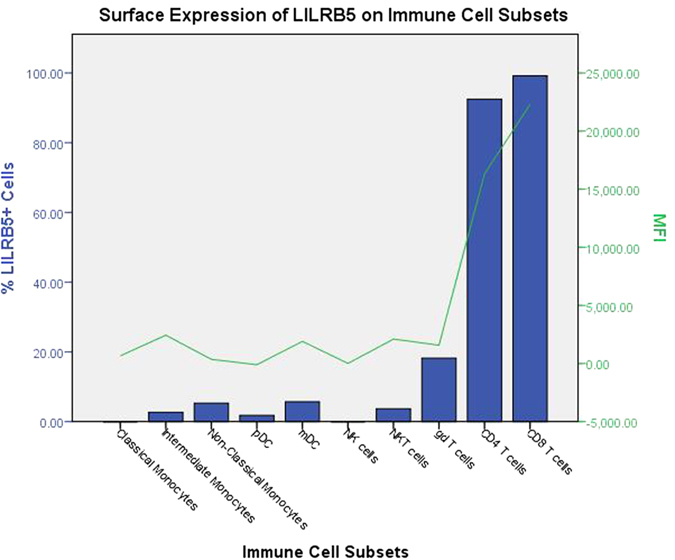
Expression summary of LILRB5 staining on immune cell subsets. This figure depicts the median percentage of each immune cell subset that stained positive for LILRB5 (n = 3), and the corresponding MFI.

To date, characterisation of LILRB5 and its functions are unclear. One investigation identified LILRB5 mRNA in NK cells^36^. In order to investigate LILRB5 surface expression more thoroughly, a commercially available LILRB5-specific antibody was used to determine the expression of LILRB5 on PBMC. A whole blood flow cytometry method was followed to analyse immune cell subsets including monocytes, pDC, mDC, NK, NKT, CD4^+^ T cells, CD8^+^ T cells and γδ T cells on cells derived from healthy controls who had received BCG vaccination (Fig. 6).

**Figure 7:**
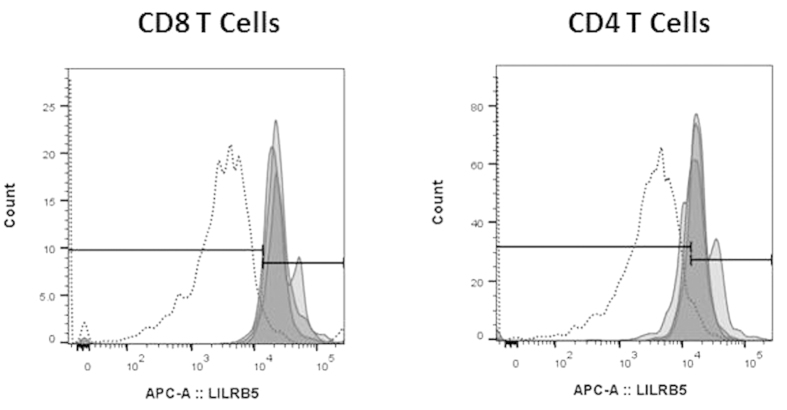
Surface expression of LILRB5 on CD4 and CD8 T cells. The secondary antibody alone was used as a control for non-specific binding. The results show the binding for three individual donors.

**Figure 8:**
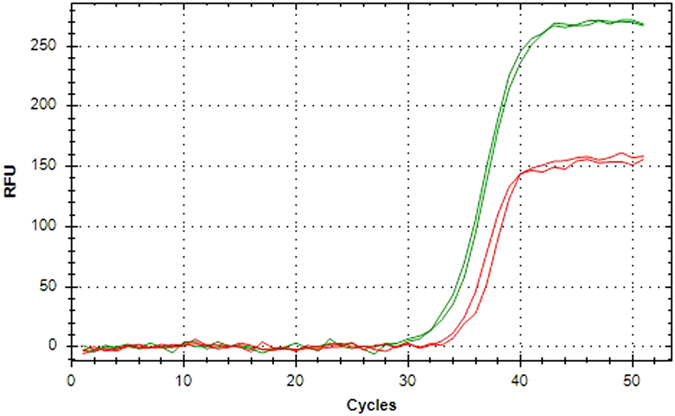
Expression of LILR encoding transcripts in T cell populations. LILRB5 amplification is represented in red, and GAPDH amplification is represented in green. LILRB5 was detected in magnetically isolated T cells from BCG vaccinated donors, using qPCR. This is a representative result from *n* = 5 samples, of which all data was consistent.

In agreement with the mRNA expression data described above, LILRB5 was expressed at very low levels on monocytes and DC. However, LILRB5 was found to be predominantly expressed on T cells (Fig. 7), and on a small percentage of γδ T cells. To confirm this data, we analysed the mRNA levels of LILRB5 for T cells isolated by magnetic separation (Fig. 8).

While we anticipated LILRB5 expression to be predominantly on antigen presenting cells (APC), with its expression on T cells somewhat unusual, LILRB1 is also known to be expressed on T cells, and thus such a precedent exists^37^.

### LILRB5 ligation increases proliferation of cytotoxic, but not helper, T cells

**Figure 9:**
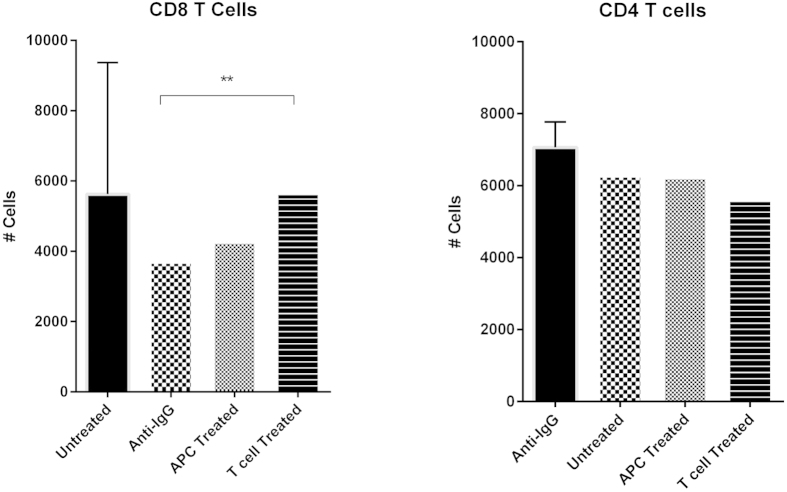
Allogeneic mixed lymphocyte reaction. Isolated CD4 of CD8 T cells, or APC, were treated with crosslinking LILRB5 antibody, and the resulting proliferation measured using flow cytometry. Crosslinking LILRB5 on CD8 T cells was found to significantly increase proliferation (p = 0.0049), as compared to the secondary antibody alone, using a two-tailed paired t test. This effect was not observed for CD4 T cells. The above data represents *n* = 4 BCG vaccinated donors.

Individual receptors in the LILR family exert functional effects that vary between cell types. The majority of studies to date have focussed on the effects of LILR activity on antigen presenting functions of monocytic cells^13^. Given the unexpected expression of LILRB5 on T cells, we investigated the potential functions of LILRB5 by crosslinking this receptor and analysing the effects using an allogeneic mixed lymphocyte reaction (Fig. 9).

Crosslinking of LILRB5 was achieved using a mouse anti-human IgG secondary antibody. However as our data shows, the secondary antibody alone consistently reduced CD8 T cell proliferation. Nonetheless, LILRB5 was able to overcome this effect, and increase proliferation when compared to the anti-IgG secondary antibody alone. Specifically, whereas APC pre-treated with LILRB5 antibody resulted a slight increase in T cell proliferation, pre-treatment of CD8 T cells produced a significant increase in proliferation compared to the secondary antibody alone (p = 0.0049). This was not found in CD4 T cell proliferation, demonstrating that, at least for cytotoxic T cells, LILRB5 ligation enhances proliferation.

While the functional relevance of increasing T cell proliferation through LILRB5 signalling needs to be further characterised before any conclusions can be drawn, cytotoxic T cells are a fundamental component of anti-mycobacterial responses, and unless functionally altered, increased proliferation may be a beneficial factor.

## Discussion

With a growing body of evidence to support the existence of ‘innate imprinting’ or ‘trained immunity’, new light is being shed on the long term effects of vaccinations, and the ways in which they might shape immune responses. Several studies have demonstrated the ability of certain proteins to permanently alter APC phenotype^38^, and BCG vaccination has been shown to increase the expression of activation markers and production of pro-inflammatory cytokines in monocytes through epigenetic reprogramming^33^,^39^. Therefore, before analysing the effects of mycobacterial infection on LILR expression, APC derived from BCG-vaccinated donors were compared with those from unvaccinated donors in order to identify any pre-existing differences in LILR expression.

While no differences were observed for moDC between the two groups, two LILR were found to differ in expression levels for MDM. LILRA3, the only member of the family to be genomically encoded as a soluble protein, was expressed at significantly lower levels in MDM from BCG vaccinated donors compared to unvaccinated donors. Similarly for LILRB2, a significantly lower expression was also observed for MDM derived from BCG vaccinated donors compared to MDM derived from unvaccinated donors.

It is interesting to note that although LILRA3 is designated as an activating receptor for the purposes of nomenclature, the functional effects of this receptor are thought to be inhibitory. This protein can only be expressed in soluble form, and soluble LILR are thought to antagonise the functions of their cell surface counterparts by blocking ligand recognition^40^. LILRA3 is thought to inhibit the interactions of the activating receptor LILRA1 in this manner^41^. Thus, our findings indicate that BCG vaccination leads to the down-regulation of two receptors with inhibitory functions, which may be a potentially beneficial response in the presence of mycobacteria.

Of all the changes detected in LILR mRNA expression, LILRB5 was the only receptor to be significantly up-regulated in response to mycobacterial challenge. In moDC, up-regulation of LILRB5 expression was exclusively seen in cells derived from BCG vaccinated donors, following exposure to mycobacteria. In MDM, only exposure to *M. bovis* elicited this up-regulation, which was again, only seen in cells derived from vaccinated donors.

Finding LILRB5 upregulated following exposure to mycobacterium species led us to hypothesise that this receptor may have a more pertinent role in infection. By incorporating a reporter system, we analysed the ability of the mycobacterial species to trigger signalling via direct engagement of transfectant cells. Our results demonstrated signalling from LILRA1 and LILRB5, indicating again a potential role for LILBR5 during infection. Furthermore, as LILRA3 is thought to inhibit LILRA1 through competitive inhibition^36^, it is interesting to note that BCG vaccination appears to reduce LILRA3 expression, possibly resulting in less inhibition of LILRA1 binding in the presence of mycoabcterial infection. Members of the LILR family and their murine orthologyes have previously been shown to bind *S.aureus* and *E.coli*^34^, and lipoteichoic acid has been identified as a putative bacterial ligand for the murine PIR-B receptor^35^. It is therefore possible to speculate that lipoteichoic acid may also be a ligand for LILRB5. However, lipoteichoic acid is heat stable^42^ and therefore its binding would not be expected to be lost following heat treatment as seen in our assays. The *M.* *tb* cell wall primarily contains mycolic acid, lipomannan, and lipoarabinomannan lipids, but these components have also been shown to be very heat stable, contributing to the robustness of this bacterium. Nonetheless, TLR-4 was shown to bind a heat-labile antigen which was referred to as ‘heat-sensitive, cell-associated factor’^26^. Further studies will be necessary to identify a mycobacterial ligand for LILRB5.

With limited published information to describe the protein expression profile, ligand specificity and function of LILRB5, it is difficult to predict whether changes in LILRB5 expression on APC might be detrimental to eliciting an effective immune response. However, LILRA3 together with LILRB5 and LILRB3 were found to be over expressed in lesions of patients with lepromatous leprosy, where their overexpression was thought to correspond to an ineffective immune response^43^. If these receptors do decrease the efficiency of immune responses it would raise the question of whether BCG vaccination does in fact prime APC in an appropriate way to generate a robust T cell response to mycobacterial infection.

LILRB5 mRNA was previously found to be expressed by NK cells^36^, while the protein has been described on the surface of osteoclasts^40^ and secreted by mast cells^44^. Other studies have documented LILRB5 up-regulation under certain conditions in adipose tissue^45^, PBMC following IFN treatment of MS^46^, TNF treated DC^47^, and malignant pleural mesothelioma^48^. LILRB5 protein was identified in an intracellular compartment of mast cells, where although it has been shown to mobilize as a result of crosslinking FcεRI, its functions remain elusive^44^.

In an attempt to further characterise this receptor, we analysed the surface expression of LILRB5 on immune subsets, using a recently available anti-LILRB5 antibody. LILRB5 was expressed by more than 80% of CD4 and CD8 T cells, and on a proportion of γδ T cells. As regulators of TLR, the role of LILR on T cells may be similar to that on APC. Whereas ligation of TLR expressed on APC results in cellular activation and maturation, TLR expression on T cells is thought to provide co-stimulatory signals during T cell activation, and promote proliferation and survival in effector cells^49^,^50^,^51^. TLR2 has also been shown to lower the activation threshold of CD8 T cells in mice^52^.

We examined the effect of cross-linking LILRB5 in an allogeneic mixed lymphocyte reaction. Pre-treatment with LILRB5 ligation significantly increased proliferation of CD8 but not CD4 T cells. It is not unusual for LILR to have differing functions depending on the type of cell they are expressed on, and therefore, whilst we can conclude that one function of LILRB5 may be to increase proliferation of cytotoxic T cells, the expression of LILRB5 on helper T cells is not associated with either promoting or inhibiting proliferation. These data would support the notion that LILRB5 expression on cytotoxic T cells may mediate the threshold of activation.

In conclusion, this study provides evidence that LILR expression and functions are affected by exposure to *M. bovis* and its attenuated strain BCG. LILRB5 is both able to engage our transfectant cells, and is up-regulated following exposure to *M. Bovis.* Further investigation revealed this receptor to be expressed predominantly on T cells, and able to enhance cytotoxic T cell proliferation. The presence of LILRB5 on T cells and its apparent ability to bind *M. bovis* warrants further investigation as to whether and how this receptor can influence the immune response during mycobacterial infection.

### Materials and Methods

#### Human Study Populations

Ethical approval for this study was granted by London and Surrey Borders Research Ethics Committee on 24^th^ May 2010, ref. 10/H0806/41, and this study was carried out in accordance with the approved guidelines. Healthy participants were recruited from St George’s, University of London, and were not selected with regard to age, sex or ethnic origin, but were asked to provide BCG vaccination details. Informed consent was obtained from all participants prior to collection of 50 ml blood samples.

#### Culture of *in vitro*-derived macrophages and dendritic cells

Peripheral blood mononuclear cells were separated using density centrifugation over Ficoll-paque (GE Healthcare, UK). Adherent cells were cultured in RPMI1640 containing 1% autologous plasma, 1% Penicillin/Streptomycin (Sigma, UK). Monocyte derived macrophages (MDM) were differentiated in 50 ng/ml M-CSF (Peprotech, UK) and monocyte derived dendritic cells (moDC) in 50 ng/ml IL-4 and GM-CSF (Peprotech, UK). Cytokines were replenished every 48 hours. After six days, 0.5 ng/ml bacterial lipopolysaccharide (LPS) (Sigma, UK) was added to mature the cells.

#### Culture of BCG, M. tuberculosis and M. bovis

Thawed aliquots of BCG, *M. tuberculosis* (H37Rv) and *M. bovis* (AF2122/97) were used to initiate cultures. Original mycobacterial aliquots were kindly provided by Prof P. Butcher (St George’s, University of London, UK) and Prof M. Vordermeier (APHA, UK). Each aliquot was cultured at 37 °C for ten days in 10mls of 7H9 broth (Difco Laboratories, UK) supplemented with 0.5% glycerol (BCG, *M. tuberculosis* (Sigma, UK)) or 0.5% sodium pyruvate (*M. bovis* (Invitrogen, UK)), followed by a further ten days in 100 mls of supplemented broth at 37 °C. Colony forming units (CFU) were assessed using 10-fold serial dilutions on 7H11 agar plates (Difco Laboratories, UK). The culture was aliquoted and frozen at −80 °C until use.

#### Infection of moDC and MDM with BCG, *M. tuberculosis* or *M. bovis*

Antibiotic-free RPMI was used for culture of cells in infection studies. Mycobacteria in the exponential phase of growth were added to cells at a multiplicity of infection (MOI) of 10:1 (*M. tuberculosis*:cells) and incubated at 37 °C for 24 hours. The cells were then washed gently three times with PBS to remove excess bacteria.

#### RNA extraction and cDNA synthesis

RNA extraction and cDNA synthesis were performed as previously described^40^. Briefly, RNA was extracted from moDC and MDM using an RNeasy Kit (Qiagen, UK) following the manufacturers standard protocol. RNA was eluted in 40 μl of 0.2 μm sterile filtered H_2_O (Sigma, UK), and underwent a second DNase treatment using a Turbo DNase Free kit (Ambion, USA). Genomic DNA contamination was assessed by quantitative PCR (qPCR) using a *GAPDH* reaction. cDNA synthesis was performed using Superscript III with an Oligo(dT)_20_ primer (Invitrogen, UK) at 50 °C for 1 hour, following the manufacturer’s standard protocol.

#### Real-Time Quantitative PCR (qPCR)

qPCR was employed to determine the relative levels of all 11 LILR transcripts in moDC and MDM. PCR primers to detect the full-length, membrane-associated protein encoding LILR transcripts were supplied by Sigma (Sigma, UK)^40^. Primer pairs, including the oligonucleotide sequences and their final working concentration were used as previously described^40^. For normalisation, reactions were also used to determine the expression of the reference gene *GAPDH*.

QPCR reactions consisted of 10 μl of 2× QuantiTect SYBR Green solution (Qiagen, UK), 1 ng cDNA, 2x oligonucleotides and brought to a final volume of 20 μl with 0.2 μm filtered H_2_O (Sigma, UK). Each sample was run in triplicate, with an accompanying non-template RNA control run in duplicate for every sample. *GAPDH* reference targets were also run for each sample in order to normalise the results. qPCR was performed on the Stratagene Mx3005 Pro PCR analyser (Stratagene, UK), or the Bio-Rad CFX96 real-time detection system (BioRad, UK). The cycling parameters were as follows: 95 °C for 15 minutes, followed by 50 cycles of 94 °C for 15 s, 60 °C for 30 s and finally 72 °C for 30 s, after which the fluorescence levels of both SYBR Green and the reference dye ROX were acquired. Baselines and thresholds were automatically calculated for all reactions on all runs using the Stratagene MxPro or CFX manager software. Finally, the standard dissociation reaction was performed between 60 °C and 95 °C in order to assess the integrity of the PCR products.

Results were exported into qBasePLUS^53^ to perform the following analyses: After calculating the average amplification efficiency (*E*) of each reaction (as assessed from three independent serial dilutions) using linear regression, the relative expression level of each target transcript in each sample was normalised against the reference gene *GAPDH*, and then the inter-plate calibrators were used to correct any inter-plate variability. Finally, the results were exported to GraphPad Prism to perform statistical analysis. Sample numbers for each analysis are shown in Table 1.

#### Determining mycobacterial binding to reporter cells expressing LILR

A cellular reporter system to assess LILR binding was generated as follows: The extracellular region of each LILR was amplified from previously cloned sequences. Each LILR PCR product was fused at the 5′ to an haemagglutinin A (HA) epitope preceded by the murine Ig k-chain leader sequence, and at the 3′ to the transmembrane domain of human platelet-derived growth factor receptor (PDGFR). These HA-tagged, LILR-PDGFR transmembrane constructs were cloned into the vector pMx puro which encoded the sequence of the cytoplasmic tail of the human CD3ζ chain (a gift from Dr Alex Barrow, St Louis USA)^54^] using the primers NK1313 (ATGCTTAATTAATCCACCATGGAGACAGAC) and NK1314 (CTTCTCGAGCCAAAGCATGATGAGGATG).

Following transient transfection of PLAT-E retroviral packaging cells with the pMx HA-LILR-PDGFR-CD3ζ fusion constructs, recombinant retrovirus was used to transduce 2B4 T cell hybridoma cells that had previously been stably transfected with a NFAT-GFP reporter construct (a kind gift from Lewis Lanier, UCSF, San Francisco, California, USA)^55^. Hereafter these cells are referred to as 2B4 reporter cells. Following transduction, 2B4 reporter cells were sorted using a MoFlo cytometer (Beckman Coulter, Brea, CA, USA) for similar levels of expression of each LILR based on the level of expression of the LILR-fused N terminal HA epitope stained with an anti-HA-PE monoclonal antibody (Miltenyi Biotec, Bergisch Gladbach, Germany).

2B4 reporter cells were cultured at 10^5^ in 200 μl RPMI (10% FCS, 1% P/S, and 2.5 μg/ml amphotericin B (PAA (Now GE Healthcare, USA)) per well of a 96 U-bottom plate. Mycobacteria were grown to exponential stage, and heat killed at 90 °C for 45 minutes. A separate experiment was performed with live BCG. Mycobacteria were added to each well at an MOI of 10:1 (*Mycobacteria*:cells) and incubated overnight at 37 °C, 5% CO_2_. The plates were then spun at 1000 rpm for one minute and the GFP level was then measured on FACScan using Weasel v2.0 software. Controls consisted of anti-HA monoclonal antibody (Miltenyi, UK) together with goat anti-mouse IgG (Fc) (ThermoScientific, UK) and cell culture only. The data was analysed using FlowJo VX.

#### Whole blood flow cytometry

Whole blood samples for flow cytometric analysis were prepared by adding the appropriate antibodies to 100 μl of whole blood, or 200 μl if diluted 1:2 with RPMI (table 2). Isotype control staining was performed for each patient and each analysis panel. Red blood cells were lysed and samples fixed using 500 μl of BD FACS lysing Solution (Becton Dickinson, UK) and incubated for up to 20 minutes. Each sample was then analysed on a BD LSR II Flow Cytometer with FACS *Diva* software (Becton Dickinson, UK).

#### Gating immune cell subsets for analysis

DC populations were identified by selecting the HLA-DR^Hi^ (PE texas red) (Invitrogen, UK) positive and Lin^−^ 1 (FITC) (Becton Dickinson, UK) negative population. Within the gated DC population, CD123^+^ (PE Cy5) (Becton Dickinson, UK) staining identified plasmacytoid DC (pDC) and CD11c^+^ (PE Cy7) (BD Pharmingen, UK) identified myeloid DC (mDC).

The monocyte population was identified using forward and side scatter characteristics and individual monocytic subsets were identified by selecting populations based on the intensity of CD16 (PE Cy7) (BD Pharmingen, UK) and CD14 (PE texas red) (Invitrogen, UK) staining, which allowed for the identification of classical, intermediate, and non-classical populations.

Lymphocyte populations were identified using forward and side scatter characteristics and T cell populations were identified by using anti-CD3 (APC-H7) (BD Bioscience), anti-CD4 (AF700) (BD Pharmingen, UK), anti-CD8 (PerCP) (BD Bioscience) and anti-γδ TCR (FITC) (BD Pharmingen, UK). Anti-CD3 antibody was plotted against anti-CD56 (PE texas red) (Invitrogen, UK) to distinguish NK cells and NKT cells.

Surface expression of LILRB5 was determined for each subset using an anti-LILRB5 primary antibody (R&D) and Anti-Mouse IgG APC (eBioscience, UK) secondary antibody.

#### Mixed Lymphocyte Reactions

Peripheral blood mononuclear cells were separated from whole blood using density centrifugation over Ficoll-paque. T cell populations were purified from non-adherent cells using a CD4 MicroBeads Kit (Miltenyi, UK) or CD8 Microbeads Kit (Miltenyi, UK) following the manufacturer’s standard protocol. LILRB5 crosslinking of APC or T cells was performed using LILRB5 antibody (R&D) crosslinked with anti-mouse IgG. Negative controls were treated with anti-mouse IgG alone, or untreated cells. Allogeneic T cells were stained using a Cell Trace Violet Proliferation Kit (Life Technologies, UK), following the manufacturer’s standard protocol, then cultured with donor APC in RPMI1640 containing 1% Penicillin/Streptomycin for four days. Proliferation was determined using flow cytometric analysis on a BD LSR II Flow Cytometer with FACS *Diva* software. Analysis was completed using FlowJo VX, by gating around the T cell population identified by forward and side scatter characteristics, and then the enumeration of T cells stained with Cell Trace Violet was determined.

#### Statistical Analysis

All statistical analyses were performed using SPSS 20 and GraphPad Prism 6.05, based on the median values for all data. Non-parametric tests were used for mRNA expression analysis, with Mann-Whitney U-test used to determine significance between unpaired groups. Parametric two-tailed T-Test was used for paired samples in the mixed lymphocyte reaction. All p-values were corrected for ties, and values below 0.05 were considered significant.

## Additional Information

**How to cite this article**: Hogan, L. E. *et al*. Expression of the innate immune receptor LILRB5 on monocytes is associated with mycobacteria exposure. *Sci. Rep.* **6**, 21780; doi: 10.1038/srep21780 (2016).

## Figures and Tables

**Table 1:** Number of donors included in each RT-PCR experiment.

	Samples derived from BCG Vaccinated donors								Samples derived from Unvaccinated donors							
	moDC				HMDM				moDC				HMDM			
	Neg^a^	BCG	M. *bovis*	H37Rv^b^	Neg^a^	BCG	M. *bovis*	H37Rv^b^	Neg^a^	BCG	M. *bovis*	H37Rv^b^	Neg^a^	BCG	M. *bovis*	H37Rv^b^
LILRA2	11	11	12	12	11	11	12	12	6	6	6	6	6	6	6	6
LILRB1	11	11	12	12	11	11	12	12	6	6	6	6	6	6	6	6
LILRB4	3	3	4	4	3	3	4	4	3	3	3	3	3	3	3	3
LILRB2	6	6	7	7	6	6	7	7	5	5	5	5	5	5	5	5
LILRB3	11	11	12	12	11	11	12	12	6	6	6	6	6	6	6	6
LILRA3	6	6	7	7	6	6	7	7	5	5	5	5	5	5	5	5
LILRA4	6	6	7	7	6	6	7	7	5	5	5	5	5	5	5	5
LILRA6	3	3	4	4	3	3	4	4	3	3	3	3	3	3	3	3
LILRA5	3	3	4	4	3	3	4	4	3	3	3	3	3	3	3	3
LILRA1	3	3	4	4	3	3	4	4	3	3	3	3	3	3	3	3
LILRB5	3	3	4	4	3	3	4	4	3	3	3	3	3	3	3	3

^a^Neg refers to untreated cells.

^b^H37Rv refers to *M. tb* strain H37Rv.

**Table 2:** Antibodies used in all experiments.

Specificity	Conjugate	Catalogue No.	Supplier
LIN 1	FITC	340546	BD
HLA-DR	PE-TR	VXMHLDR17	Invitrogen
CD11c	PE-Cy7	561356	BD Pharmingen
CD123	PE-Cy5	561009	BD
CD14	PE-TR	MHCD1417	Invitrogen
CD16	Pe-Cy7	557744	BD Pharmingen
CD3	APC-H7	641397	BD Bioscience
CD4	AF700	560836	BD Pharmingen
CD8	AF700	557945	BD Pharmingen
γδ TCR	FITC	561995	BD Pharmingen
CD56	PE-TR	VXMHCD5617	Invitrogen
LILRB5/LIR8	Purified	MAB3065	R&D
Anti-mouse IgG	APC	17-4012-82	Ebioscience
Anti-HA	PE	130-092-257	Miltenyi
Anti-Mouse IgG	PE	31861	Thermo
Mouse IgG2b	FITC	11-4732-41	Ebioscience
Mouse IgG1	APC	550854	BD
Mouse IgG_1_, κ	APC-H7	561427	BD
Mouse IgG2a	PE-Cy7	552868	BD
Mouse IgG2a	PE-TR	MG2a17	Invitrogen
